# *Candida albicans*-Based Immune Training to Reduce Lung Fibrosis from Polystyrene Nanoparticles

**DOI:** 10.3390/ijms27104169

**Published:** 2026-05-07

**Authors:** Zhuyun Zhu, Yifan Zhou, Dongnan Zheng, Zemin Xu, Yuqing Chen, Fengxu Wang, Xinyuan Zhao, Yuanyuan Ma

**Affiliations:** Nantong Key Laboratory of Environmental Toxicology, Department of Occupational Medicine and Environmental Toxicology, School of Public Health, Nantong University, Nantong 226019, China

**Keywords:** *Candida albicans*, immune training, microplastics, PS-NPs, lung fibrosis

## Abstract

Polystyrene nanoparticles (PS-NPs) present significant risks to respiratory health, contributing to lung fibrosis. Current therapeutic strategies for PS-NP exposure injuries are limited and often ineffective. One promising solution is immunological training. This study explores the novel role of *Candida albicans* in immune training to alleviate PS-NP-induced fibrosis. Our findings demonstrate that *C. albicans* enhances immune responses in a unique way, advancing current frameworks of trained immunity and offering new therapeutic approaches for health issues related to environmental pollutants. We conducted experiments using male BALB/c mice exposed to 80 nm PS-NPs via posterior pharyngeal drip. Prior to exposure, the mice received an intravenous injection of low-dose *C. albicans* to induce immunological training. To evaluate the protective effects of *C. albicans*, we assessed survival rates, pulmonary histopathology, and gene expression profiles. The results indicated that while 100% of the control group exposed to PS-NPs did not survive, the group pre-treated with *C. albicans* exhibited complete survival. Histopathological analysis revealed preserved lung architecture and a significant reduction in collagen deposition in the *C. albicans* + PS-NPs group compared to the PS-NPs only group. Additionally, RNA sequencing analysis identified a total of 415 differentially expressed genes, including five upregulated circadian rhythm genes (*Per3*: 1.93-fold, *Rorc*: 1.69-fold, *Cry1*: 2.01-fold, *Per1*: 2.55-fold, *Per2*: 2.73-fold) and one downregulated circadian rhythm gene (*Npas2*: 0.42-fold) in the *C. albicans* + PS-NPs group compared to the PS-NPs group. *C. albicans*-based immune training reduces lung fibrosis and enhances survival after PS-NP exposure, suggesting a promising therapeutic strategy.

## 1. Introduction

Microplastic pollution, especially from polystyrene nanoparticles (PS-NPs), presents a considerable global environmental and health challenge [1]. Due to their nanoscale dimensions, PS-NPs can penetrate biological barriers and invade the respiratory system, leading to accumulation in the lungs. This exposure may trigger oxidative stress and engage vital cellular pathways, including ferroptosis and TGF-β signaling, both of which are tightly linked to chronic inflammation and cellular injury. Specifically, PS-NPs can induce prolonged endoplasmic reticulum stress and mitochondrial dysfunction in alveolar epithelial cells, resulting in cellular disturbances that enhance the release of damage-associated molecular patterns. This release activates the NLRP3 inflammasome, contributing to the development of a pro-fibrotic microenvironment. Consequently, these processes may give rise to irreversible pathological changes, such as pulmonary fibrosis [2,3,4,5]. Currently, corticosteroids represent the primary clinical approach for managing these injuries; however, they provide only temporary symptom relief and do not prevent disease progression. Moreover, corticosteroid use is associated with adverse side effects, including delayed wound healing and an increased risk of infections [6,7,8]. These limitations underscore the urgent need to explore alternative therapeutic strategies.

In this regard, immunological training has emerged as a promising approach. For example, pre-treatment with β-glucan has been demonstrated to induce sustained functional reprogramming of innate immune cells via epigenetic and metabolic remodeling, resulting in enhanced protection against subsequent inflammatory challenges in experimental models [9,10]. *Candida albicans*, a common commensal fungus in the human microbiome, typically remains benign under conditions of immune homeostasis. However, it can become an opportunistic pathogen when the host’s immune function is compromised [11,12]. Research indicates that specific microbial stimuli can elicit sustained functional reprogramming in innate immune cells, a phenomenon known as “trained immunity,” which bolsters the host’s non-specific defense against diverse forms of harm [13,14]. This mechanism has been demonstrated through interventions such as the BCG vaccine, showing that moderate immune activation can prompt long-lasting protective responses [15,16,17]. In this study, we investigated the impact of low-dose *C. albicans* pre-treatment on pulmonary fibrosis and survival outcomes in mice exposed to PS-NPs using appropriate animal models.

## 2. Results

### 2.1. C. albicans Immune Training Reduces PS-NPs-Induced Lung Fibrosis

**Figure 1:**
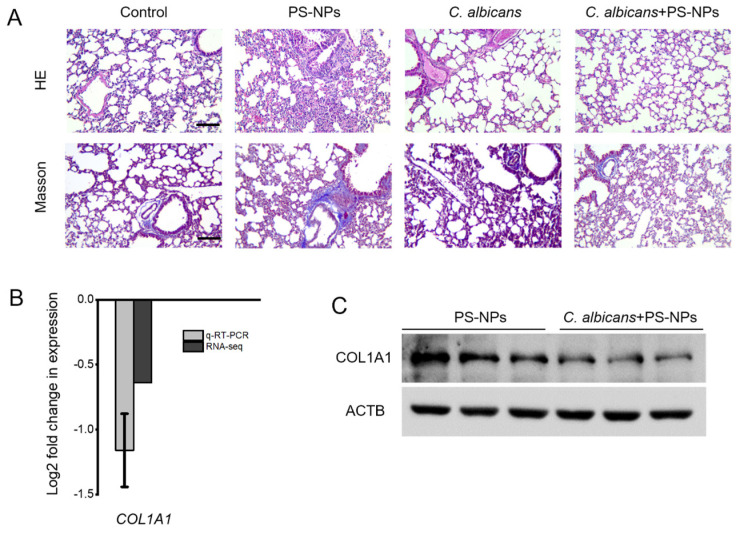
Immune training with *C. albicans* reduces lung fibrosis induced by PS-NPs in mice. The Control group received a tail vein injection of 100 μL of sterile PBS on Day 1, followed by a posterior pharyngeal drip of 50 μL of sterile PBS three times weekly. The PS-NPs group received an initial tail vein injection of 100 μL of sterile PBS and a nasal administration of 50 μL of PS-NPs three times weekly. The *C. albicans* group was administered a tail vein injection of 100 μL of PBS containing 20 *C. albicans* cells, along with a nasal administration of 50 μL of sterile PBS three times weekly. Lastly, the *C. albicans* + PS-NPs group received 100 μL of PBS with 20 *C. albicans* cells and a posterior pharyngeal drip of 50 μL of PS-NPs, applied three times weekly for the same duration. Lung tissues were analyzed after 28 days of treatment using HE staining and Masson’s trichrome staining, with a scale bar of 250 μm (**A**). qRT-PCR and RNA-Seq were used to compare transcript levels of the *COL1A1* gene between the PS-NPs and *C. albicans* + PS-NPs groups. For qRT-PCR, fold changes were calculated using the ΔΔCt method and log2 transformed, with statistical significance defined as *p* < 0.05 and a fold change threshold of >1.5 or <0.67. In the RNA-Seq analysis, the same *p*-value threshold and fold change criteria were applied prior to log2 transformation (**B**). COL1A1 protein expression was compared between the PS-NPs and *C. albicans* + PS-NPs groups using Western blot analysis (**C**).

We evaluated the effect of low-dose *C. albicans* immune training on lung fibrosis induced by PS-NPs. In the mouse model, HE staining showed that the alveolar septa in the PS-NPs group were significantly thickened, indicating lung damage. In contrast, the lung structure of the *C. albicans* + PS-NPs group remained significantly intact (Figure 1A). Masson trichrome staining further indicated that collagen deposition in the lung tissue of the *C. albicans* + PS-NPs group was significantly reduced, suggesting alleviation of fibrotic changes (Figure 1A). At the molecular level, *C. albicans* pretreatment significantly decreased the expression of *COL1A1*, a key fibrosis marker. qRT-PCR and RNA-seq analyses showed that the *COL1A1* mRNA levels in the *C. albicans* + PS-NPs group were significantly lower than those in the PS-NPs group (Figure 1B). This result was supported by Western blot analysis, which also showed reduced COL1A1 protein levels in the *C. albicans* + PS-NPs group (Figure 1C and Supplementary Figures S2 and S3). Importantly, *C. albicans* was not detected in the blood of the *C. albicans* and *C. albicans* + PS-NPs groups (Supplementary Figure S1), indicating that the immune training effect was achieved without systemic dissemination. Gene symbols reported in this study follow the NCBI Gene database annotation (https://www.ncbi.nlm.nih.gov/gene, accessed on 1 May 2026). The male BALB/c mice used in this study are genetically characterized in the Mouse Genome Informatics database (MGI, https://www.informatics.jax.org). Overall, these data emphasize the effectiveness of *C. albicans* immune training in alleviating lung fibrosis associated with PS-NP exposure.

### 2.2. Immunomodulatory Effects of C. albicans Immune Training on PS-NPs-Induced Pulmonary Fibrosis

**Figure 2:**
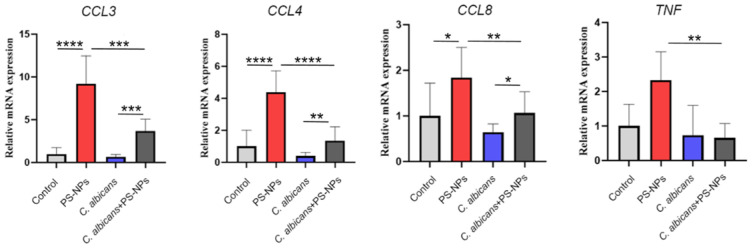
Assessment of lung mRNA expression levels of *CCL3*, *CCL4*, *CCL8*, and *TNF* via qRT-PCR. Statistical significance was evaluated using the *t*-test: * *p* < 0.05, ** *p* < 0.01, *** *p* < 0.001, **** *p* < 0.0001.

**Figure 3:**
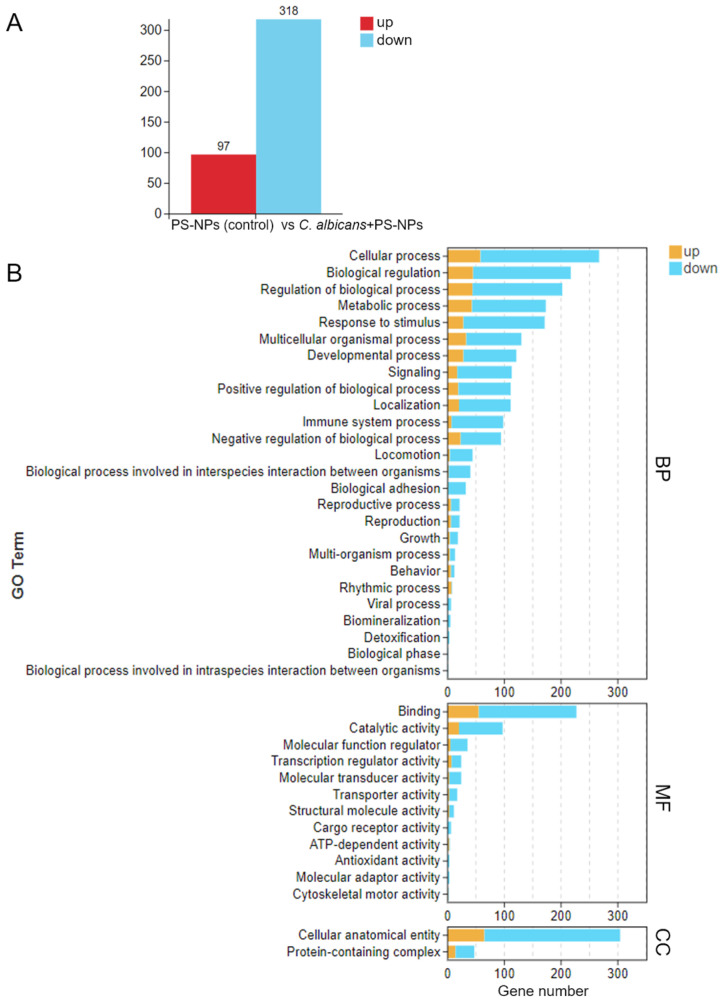
Impact of *C. albicans* immune training on lung transcriptional regulation following PS-NPs treatment in mice. A total of 97 genes were upregulated (red) and 318 genes were downregulated (blue) in the lungs after 28 days of treatment, comparing the PS-NPs group to the *C. albicans* + PS-NPs group (**A**). GO categories were assigned to the differentially expressed genes identified through RNA-seq analysis of lung tissue. BP: Biological Process; MF: Molecular Function; CC: Cell Component (**B**).

**Figure 4:**
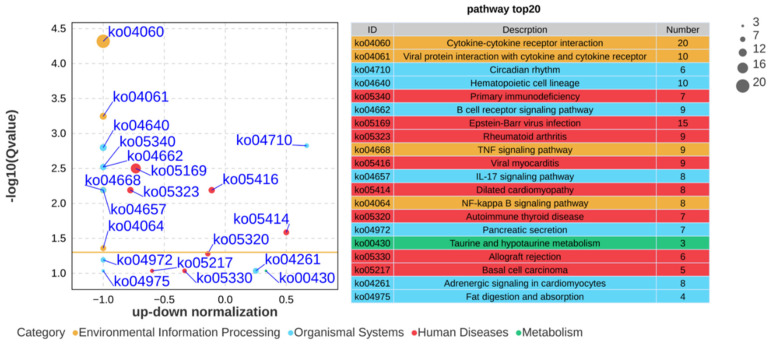
The KEGG enrichment analysis reveals the top 20 enriched pathways for differentially expressed genes between the PS-NPs group and the *C. albicans* + PS-NPs group. A bubble plot depicting the KEGG enrichment of differentially expressed genes. The vertical axis displays −log10(Q-value), while the horizontal axis represents the z-score, calculated as the difference between the counts of upregulated and downregulated genes relative to the total number of differentially expressed genes. The yellow line marks the threshold at Q-value = 0.05. On the right side, there is a list of the 20 pathways with the lowest Q-values.

To elucidate how *C. albicans* immune training provides protection against PS-NP-induced lung fibrosis, we measured the mRNA expression levels of key inflammatory cytokines (*CCL3*, *CCL4*, *CCL8*, and *TNF*) and compared the control, PS-NPs, *C. albicans*, and *C. albicans* + PS-NPs groups. The results indicated no significant differences in the lung mRNA expressions of *CCL3*, *CCL4*, *CCL8*, and *TNF* between the control and *C. albicans* groups. However, compared to the *C. albicans* + PS-NPs group, the expression levels of these cytokines were significantly increased in the PS-NPs group. Additionally, analyses showed that *CCL3*, *CCL4*, and *CCL8* were significantly upregulated in the PS-NPs group compared to the control group (Figure 2). These results suggest that *C. albicans* treatment effectively mitigated the inflammatory response associated with PS-NP exposure. To further explore the molecular differences between the PS-NPs and *C. albicans* + PS-NPs groups, we conducted RNA-seq analysis and identified 415 differentially expressed genes, including 97 upregulated genes and 318 downregulated genes (Figure 3A). Gene ontology analysis revealed significant downregulation of genes associated with various biological processes, molecular functions, and cellular components, with the number of downregulated genes exceeding that of upregulated genes. However, in the category of rhythmic process, the number of upregulated genes surpassed the downregulated genes (Figure 3B). KEGG pathway analysis further confirmed several downregulated pathways, including cytokine-cytokine receptor interaction, viral protein interaction with cytokines and their receptors, primary immunodeficiency, B cell receptor signaling pathway, Epstein–Barr virus infection, rheumatoid arthritis, and TNF signaling pathway (Figure 4). Notably, the circadian rhythm was found to be upregulated. To validate our RNA-seq results, we performed qRT-PCR analysis on selected genes, confirming that the *CCL3* mRNA levels were significantly lower in the *C. albicans* + PS-NPs group compared to the PS-NPs group (Supplementary Figure S4). These findings highlight the protective role of *C. albicans* immune training in alleviating lung fibrosis and suggest a potential involvement of both inflammatory and circadian signaling pathways, warranting further investigation.

### 2.3. C. albicans Immune Training Reduces Mortality Induced by PS-NPs

**Figure 5:**
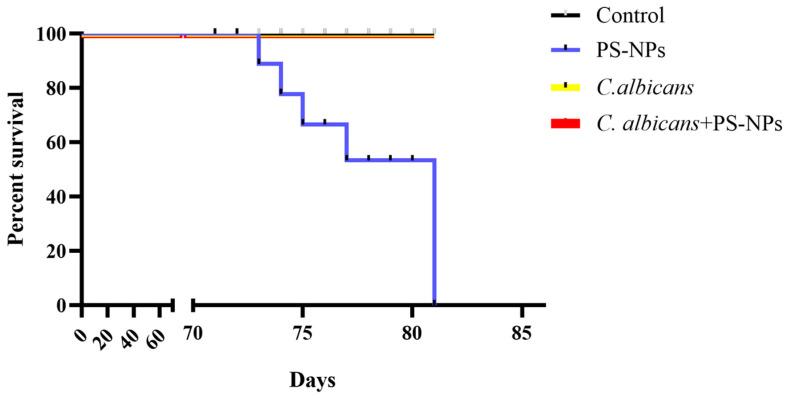
Immune training with *C. albicans* reduces mortality in mice caused by PS-NPs. Survival analysis was performed over 81 days with the four treatment groups. The log-rank Mantel–Cox test was utilized to analyze survival differences, with each group consisting of 8 mice.

We conducted a comprehensive analysis of survival outcomes following PS-NP exposure. The results were striking: the PS-NPs group exhibited a 100% mortality rate, underscoring the severe toxicity of these nanoparticles. In contrast, the control, *C. albicans*, and *C. albicans* + PS-NPs groups exhibited significant resilience, with no recorded deaths throughout the observation period (Figure 5). This complete protection from lethal outcome highlights the potent role of *C. albicans* as an effective immune training agent.

## 3. Discussion

In this study, we investigated the protective effects of low-dose *C. albicans* immune training against mortality and lung fibrosis induced by PS-NPs in a murine model. Our findings demonstrate that the pre-treatment with *C. albicans* significantly enhances survival rates in mice exposed to PS-NPs, with the *C. albicans* + PS-NPs group showing complete protection (no recorded deaths) over an 81-day observation period. The histopathological analyses revealed significantly better-preserved lung architecture and reduced fibrotic changes in the *C. albicans* + PS-NPs group compared to the control group. Furthermore, molecular analyses indicated lower expression levels of COL1A1, a key marker for fibrosis. Collectively, these results underscore the remarkable efficacy of *C. albicans* immune training in counteracting the severe pulmonary toxicity of PS-NPs.

Our findings are further substantiated by comparisons with previous studies. Prior research has indicated that low-dose microbial exposures, such as those involving Mycobacterium bovis BCG or β-glucan, can elicit trained immunity, thereby providing protection against various unrelated inflammatory insults [18]. Furthermore, our results are consistent with studies demonstrating that fungal components can mitigate fibrosis in models of bleomycin-induced lung injury [19]. Notably, to the best of our knowledge, this study is the first to demonstrate that live, low-dose *C. albicans* training confers protection against nanoparticle-induced pulmonary fibrosis. This expands the concept of trained immunity into the realm of environmental nanosized pollutants.

The efficacy of *C. albicans* in promoting positive health outcomes raises pertinent questions about the underlying mechanisms responsible for the protective effects observed in the *C. albicans* + PS-NPs group compared to the PS-NPs group. Our data suggest that the immune system was activated locally without systemic dissemination of *C. albicans*. This localized immune activation likely facilitated a balanced immune response, thereby alleviating the detrimental effects associated with PS-NP exposure. Histopathological assessments further corroborate this conclusion. The maintained lung architecture, as evidenced by HE staining, alongside significantly reduced collagen deposition shown in Masson’s trichrome staining, indicates a controlled inflammatory response. In contrast, the PS-NPs group exhibited marked alveolar septal thickening and significant lung injury, both indicative of an unregulated immune response. Additionally, our RNA-seq analysis revealed that genes associated with circadian rhythm were upregulated in the *C. albicans* + PS-NPs group. This finding provides a preliminary clue for further investigation. Circadian rhythms are well-established modulators of immune responses [20,21,22], and their influence extends to the bidirectional regulation of the nervous and endocrine systems. Notably, changes in cytokine balance due to immune training can impact the activity of the hypothalamic–pituitary–adrenal axis, consequently altering corticosterone release. This hormonal shift can feed back to modify immune cell function and circadian gene expression [23]. Conversely, neuroendocrine signals, such as melatonin and cortisol, directly influence the expression of circadian clock genes (e.g., *Clock*, *Bmal1*) in immune cells, thereby regulating the production of inflammatory cytokines [24]. In our proposed model, the upregulation of circadian pathway genes may signify an integrated immune-neuroendocrine adaptation aimed at mitigating excessive fibrosis. However, the precise contribution of this pathway to the observed protective effects remains to be experimentally elucidated. Therefore, we propose a testable hypothesis: Immune training by *C. albicans* may provide protection by orchestrating a balanced inflammatory response, potentially through the modulation of circadian signaling networks and their interactions with neuroendocrine pathways.

Beyond immune modulation, *C. albicans* may bolster resilience against environmental stressors through various immune training mechanisms. Prior research has indicated that low-dose microbial exposure can induce trained immunity, leading to functional reprogramming of innate immune cells and enhancing their preparedness to respond to subsequent threats. This phenomenon could explain the improvements in survival and reduced fibrosis observed in our study, as a trained immune system is better equipped to manage the inflammatory challenges posed by PS-NPs. Additionally, an alternative mechanism worth considering, which is not mutually exclusive, is that low-dose pre-treatment with *C. albicans* may partially “distract” the immune system. This distraction occurs by engaging innate immune receptors such as Dectin-1 and TLR2, leading to a controlled, low-grade immune response. By redirecting the immune system’s focus in this way, it could prevent the overwhelming and dysregulated immune activation that is typically elicited by PS-NPs alone, thus minimizing collateral tissue damage and fibrosis. This phenomenon of “immune distraction” has been proposed in other models of heterologous protection [25] and should be explored further in the context of nanoparticle toxicity.

However, the use of live *C. albicans* for immune training poses potential risks, including the threat of uncontrolled infections or paradoxical exacerbation of inflammation, particularly in immunocompromised individuals. Thus, the initial immune status of the host and the specific dosing regimen are critical factors that influence both safety and efficacy. Despite these risks, we hypothesize that low-dose pre-treatment with *C. albicans* may offer an immune training strategy by promoting epigenetic reprogramming and metabolic shifts in innate immune cells. This could enhance both local and systemic innate immune memory, thereby increasing pulmonary resistance to inflammation and fibrotic processes triggered by subsequent exposure to PS-NPs, ultimately conferring a protective effect. In summary, while our study presents compelling evidence for the profound protective effects of *C. albicans* immune training, the precise mechanisms—particularly the role of circadian pathways—remain an open question. Further mechanistic studies, including targeted gene knockouts or pharmacological interventions, are essential to dissect these complex interactions.

## 4. Materials and Methods

### 4.1. Strain and Media

The strain of *C. albicans* used in this study was the standard wild-type strain SC5314. It was cultured on yeast extract, peptone, and dextrose medium (YPD) at 30 °C under shaking conditions (130 rpm) to ensure optimal growth.

### 4.2. PS-NPs

The PS-NPs (80 nm) were obtained from the Base Line Chrom Tech Research Centre in Tianjin, China. To ensure a uniform suspension, these nanoplastics were subjected to ultrasonication for 30 min prior to administration. The characterization and dosing details (5 μg/μL, 50 μL, administered three times a week) of the PS-NPs (80 nm), as well as the exposure duration, were described in our previous publications [2,3,26].

### 4.3. Mouse Models

In this study, male BALB/c mice aged 6 to 8 weeks were sourced from the Experimental Animal Center at Nantong University in Nantong, China, to evaluate the immune training effects of *C. albicans* on lung fibrosis induced by PS-NPs. Wild-type *C. albicans* cells were cultured overnight in YPD medium, washed with phosphate-buffered saline (PBS), and prepared for injection. Four experimental groups were established. The Control group consisted of eleven mice that received a single tail vein injection of 100 μL sterile PBS, followed by posterior pharyngeal drip administration of 50 μL sterile PBS starting one day post-injection. This administration was performed three times a week on Tuesdays, Thursdays, and Saturdays over a total observation period of 81 days to simulate chronic inhalation exposure. The PS-NPs group also included eleven mice that received the same tail vein injection of PBS, along with posterior pharyngeal drips of 50 μL PS-NPs suspension (5 μg/μL, 80 nm) starting one day after the initial injection, following the same schedule as the Control group. In the *C. albicans* group, eleven mice were administered a single tail vein injection of PBS containing 20 *C. albicans* cells, followed by posterior pharyngeal drip administration of 50 μL sterile PBS commencing the following day, adhering to the same drip schedule. Lastly, the *C. albicans* + PS-NPs group included eleven mice that received a single treatment with PBS containing *C. albicans* cells, along with posterior pharyngeal drip administration of 50 μL PS-NPs suspension (5 μg/μL, 80 nm) starting one day post-injection, similar to the other groups. After an initial treatment period of 28 days, three randomly selected mice from each group were euthanized via CO_2_ inhalation for analysis. Lung tissues from the euthanized mice that underwent the 28-day treatment were collected for further analysis, including hematoxylin-eosin (HE) staining, Masson’s trichrome staining, Western blot analysis, quantitative reverse transcription PCR (qRT-PCR), and RNA sequencing (RNA-Seq). To assess the presence of *C. albicans* in the bloodstream, blood samples from the 28-day treated mice were homogenized in 1 mL of PBS using a MixerMill (Retsch, Haan, North Rhine-Westphalia, Germany). The homogenates underwent serial dilutions and were subsequently plated on YPD agar plates supplemented with 35 µg/mL chloramphenicol. Colony-forming units were determined after 3 days of incubation at 30 °C. The remaining eight mice continued with the posterior pharyngeal drip treatment for a total observation period of 81 days, during which their mortality rate was systematically monitored. Daily health assessments were conducted on the mice, and those that reached the humane endpoint were euthanized using CO_2_ asphyxiation, strictly following the ethical guidelines set forth by Nantong University to minimize suffering.

### 4.4. Histopathology

Lung tissues were fixed using 4% paraformaldehyde and embedded in paraffin blocks. Sections of 4 μm were then cut and mounted on slides. For the assessment of histopathological alterations, HE staining was conducted in accordance with the manufacturer’s instructions. Additionally, Masson staining was utilized to examine the fibrotic regions present in the lung sections.

### 4.5. Western Blot Analysis

Lung tissue proteins were extracted using RIPA lysis buffer (Beyotime, #P1046, Shanghai, China), supplemented with protease inhibitors to maintain protein integrity. The concentration of protein in each sample was assessed with the bicinchoninic acid (BCA) assay (Beyotime, #P0009, Shanghai, China). An approximate amount of 30 μg of protein from each sample was resolved by SDS–PAGE on either a 10% or 15% gel. After electrophoresis, the proteins were transferred to a polyvinylidene difluoride (PVDF) membrane (Millipore Corporation, Billerica, MA, USA, #MA 01821), which was subsequently blocked at room temperature with 5% skim milk to prevent non-specific binding. The membrane was then incubated overnight at 4 °C with anti-COL1 antibody (1:1000; Proteintech, Rosemont, IL, USA, #14695-1-AP) and rabbit anti-β-actin antibody (1:1000; Sigma, St. Louis, MO, USA, #sc-69879). Following this incubation, a secondary antibody (goat anti-rabbit IgG from Sigma, St. Louis, MO, USA, #A0545) was applied at room temperature for one hour at a dilution of 1:20,000. Visualization of the protein bands was achieved using the Tanon-5200 imaging system (Tanon Company, Shanghai, China). The intensity of these bands was analyzed and quantified with ImageJ software (version 1.54) (National Institutes of Health, Bethesda, MD, USA), utilizing β-actin as a normalization reference.

### 4.6. RNA-Seq Analysis

Total RNA was extracted from the lungs of mice after a 28-day treatment in both the PS-NPs group and the *C. albicans* + PS-NPs group, utilizing TRIzol reagent (catalog #15596026, Invitrogen, Carlsbad, CA, USA). The concentration and purity of the RNA were determined with the Nanodrop 2000 (Eppendorf, Barkhausenweg, Hamburg, Germany), while its integrity was assessed through gel electrophoresis and the Agilent 2100 system (Bio-Rad, Hercules, CA, USA). Indexed RNA-Seq libraries were constructed using 800 ng of total RNA from the TruSeq RNA Library Prep Kit v2 by Illumina (Illumina, San Diego, CA, USA). Sequencing of the libraries was performed on an Illumina HiSeq2500 platform by Gene Denovo Biotechnology Co. (Guangzhou, China). The sequencing reads were aligned to the reference genome using the Bowtie program and tools (version 1.3.1) for hierarchical indexing of spliced alignment from the NCBI assembly database. Transcript abundances were calculated using the RNA-Seq by expectation-maximization method, reporting values in fragments per kilobase per million mapped reads. Differentially expressed genes were identified by setting a threshold of *p* < 0.05, along with a fold change exceeding 1.5 or falling below 0.67. R software (version 4.2.2) was employed to visualize the expression data, including a volcano plot generated with the ggplot2 package (version 4.0.0). All DEGs were associated with Gene Ontology (GO) terms from the GO database [27], and the number of genes linked to each term was compiled. Significant GO terms enriched among the DEGs compared to the genome background were identified using a hypergeometric test. For the enrichment analysis, functional annotations were derived from the Kyoto Encyclopedia of Genes and Genomes (KEGG) pathway databases [28].

### 4.7. qRT-PCR

RNA was isolated from snap-frozen lung tissue utilizing the Trizol reagent from Takara, Japan. Subsequent to this, cDNA synthesis was performed with a reverse transcription kit also provided by Takara, following established protocols. The quantification of *COL1A1*, *CCL3*, *CCL4*, *CCL8*, and *TNF* gene expression in the lung samples was conducted using RT-PCR on a Roche LightCycler 480 system (Roche Diagnostics GmbH, Mannheim, Baden-Württemberg, Germany). To ensure accurate measurement of the target genes’ relative expression, the GAPDH gene functioned as the internal control. The relative expression levels were determined through the 2^−ΔΔCt^ method, with three technical replicates included for reliability. Primers were created based on sequences from the NCBI database and obtained from Bioligo (Shanghai, China). The details of the primer sequences are available in Supplementary Table S1.

### 4.8. Statistical Analysis

All data were analyzed using OriginPro 2020 or GraphPad Prism 9.0 and are expressed as mean ± standard deviation (SD). Comparisons between two groups were conducted using a two-tailed Student’s *t*-test. For survival analysis, the log-rank Mantel–Cox test was utilized. A *p*-value of less than 0.05 was considered statistically significant (*p* < 0.05, * *p* < 0.01, ** *p* < 0.001, *** *p* < 0.0001).

## Data Availability

The RNA-Seq data can be accessed in the Genome Sequence Archive (GSA: CRA036655) at the following link: https://ngdc.cncb.ac.cn/gsa, accessed on 7 January 2026.

## Supplementary Materials

The following supporting information can be downloaded at https://www.mdpi.com/article/10.3390/ijms27104169/s1.
