# Supplementary material for: Candida albicans-Based Immune Training to Reduce Lung Fibrosis from Polystyrene Nanoparticles
Source: Int J Mol Sci. 2026 May 7;27(10):4169. doi: 10.3390/ijms27104169 (PMC13206347; doi:10.3390/ijms27104169)
Supplement: Supplementary file 1 [file ijms-27-04169-s001.zip › ijms-4296558-supplementary.pdf]

Supplementary Figures and Tables

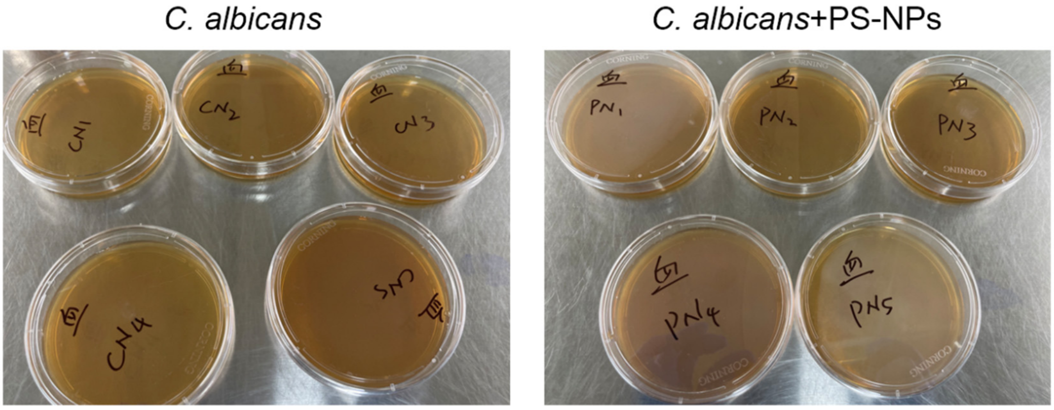

Supplementary Figure S1. Evaluation of C. albicans presence in the blood of mice. The presence of *C. albicans* in the blood of either the *C. albicans* group or the *C. albicans* + PS-NPs group was assessed through blood samples collected 28 days after a tail vein injection of *C. albicans*. The blood samples were plated on YPD media and incubated at 30°C for 3 days.

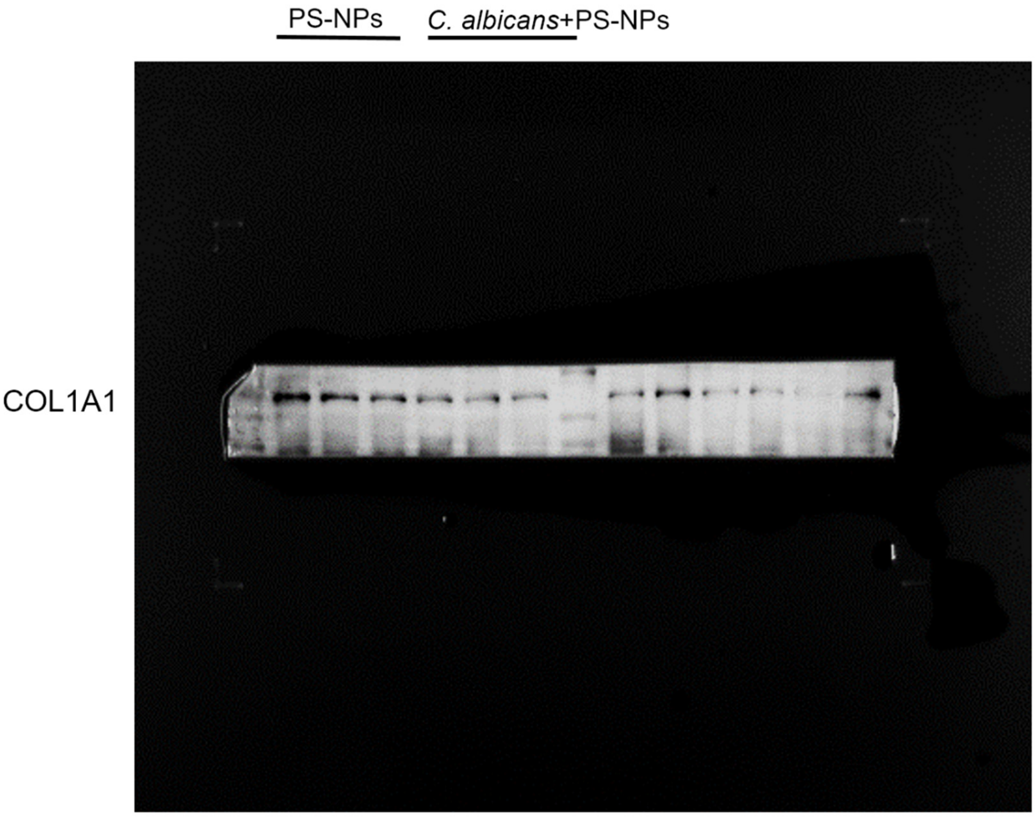

Supplementary Figure S2. Original Western blot images of the COL1A1 protein.

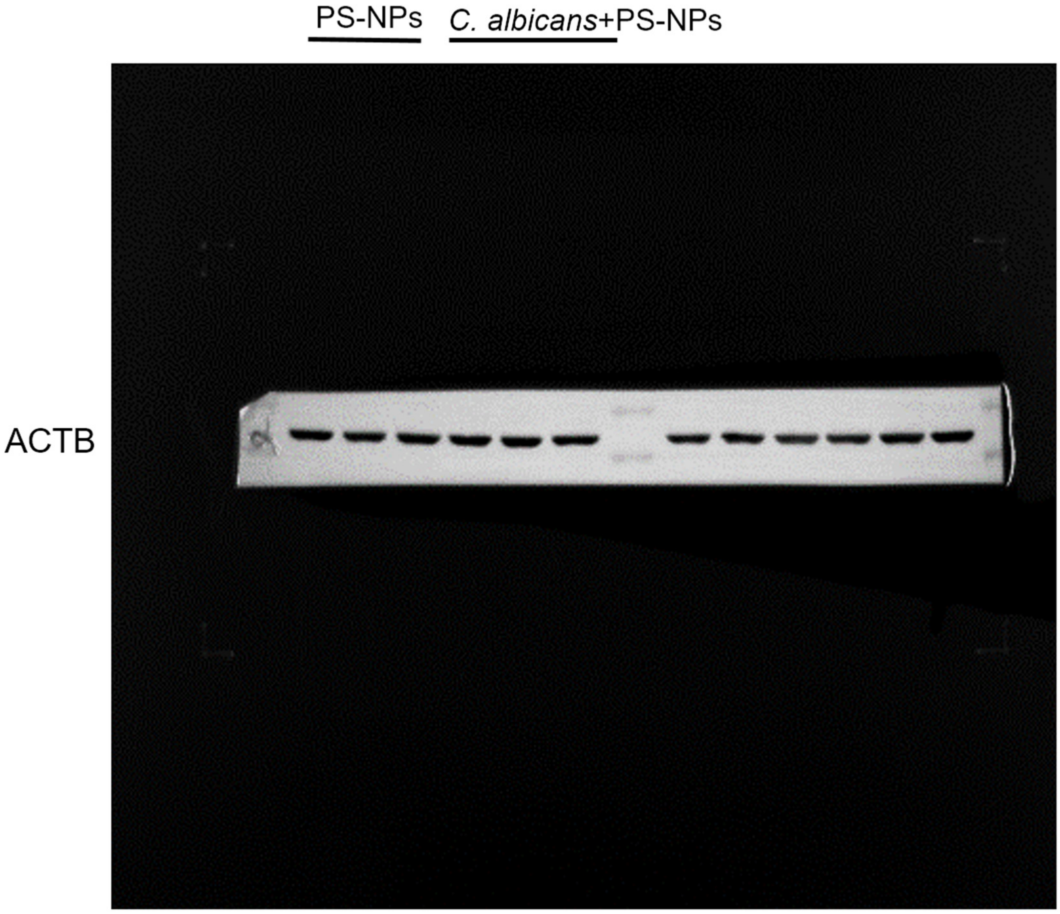

Supplementary Figure S3. Original Western blot images of the ACTB protein.

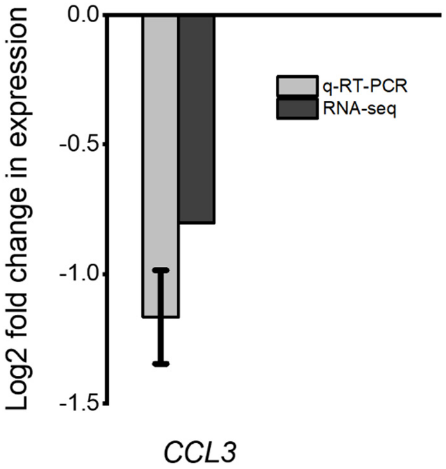

Supplementary Figure S4. CCL3 gene transcript levels were compared between PS-NPs and *C. albicans*+PS-NPs groups using RNA-Seq and qRT-PCR. Significance was defined as P < 0.05 with a fold change > 1.5 or < 0.67.

Supplementary Table S1 Primer sequences utilized for qRT-PCR. F: forward primer; R: reverse primer.

Name	Sequence 5'-3'	Reference
COL1A1_F	gcaagaggcgagagaggttt	This Study
COL1A1_R	gaccacgggcaccatcttta	This Study
CCL3_F	gcttctcctacagccggaag	This Study
CCL3_R	aggtctctttggagtcagcg	This Study
CCL4_F	gccagctgtggtattcctga	This Study
CCL4_R	ctcatgtactcagtgacccagg	This Study
CCL8_F	gccagataaggctccagtca	This Study
CCL8_R	tccatggggcactggatattg	This Study
TNF_F	gatcggtccccaaagggatg	This Study
TNF_R	ctacgacgtgggctacagg	This Study
